# Supplementary material for: Effectiveness of Inactivated COVID-19 Vaccines against Delta-Variant COVID-19: Evidence from an Outbreak in Inner Mongolia Autonomous Region, China
Source: Vaccines (Basel). 2023 Jan 28;11(2):292. doi: 10.3390/vaccines11020292 (PMC9961555; doi:10.3390/vaccines11020292)
Supplement: Supplementary file 1 [file vaccines-11-00292-s001.zip › vaccines-2141376-supplementary.pdf]

Supplementary file

Table S1. Characteristics of all investigated close contacts by COVID-19 vaccination history (No., %)

Age(years)	**Total**	**0-doses**	**1-dose**			**2-dose**				**3-dose**	
			**0-14 days ***	**15-90 days**	**>90 days**	**0-14 days**	**15-90 days**	**91-180 days**	**>180 days**	**0-6 days**	**≥7 days**
Total	10788	644	761	128	68	16	605	4455	2168	388	1555
3-11	1028	227 (22.1)	733 (71.3)	68 (6.6)	0 (0)	0 (0)	0 (0)	0 (0)	0 (0)	0 (0)	0 (0)
12-17	896	14 (1.6)	25 (2.8)	14 (1.6)	6 (0.7)	6 (0.7)	163 (18.2)	668 (74.6)	0 (0)	0 (0)	0 (0)
18-59	7740	248 (3.2)	1 (0)	39 (0.5)	55 (0.7)	9 (0.1)	309 (4)	3150 (40.7)	2064 (26.7)	364 (4.7)	1501 (19.4)
≥60	1124	155 (13.8)	2 (0.2)	7 (0.6)	7 (0.6)	1 (0.1)	133 (11.8)	637 (56.7)	104 (9.3)	24 (2.1)	54 (4.8)
Positive	503	39	14	7	4	0	30	269	97	7	36
3-11	24	7 (29.2)	13 (54.2)	4 (16.7)	0 (0)	0 (0)	0 (0)	0 (0)	0 (0)	0 (0)	0 (0)
12-17	104	1 (1)	0 (0)	0 (0)	1 (1)	0 (0)	12 (11.5)	90 (86.5)	0 (0)	0 (0)	0 (0)
18-59	290	12 (4.1)	1 (0.3)	2 (0.7)	3 (1)	0 (0)	12 (4.1)	133 (45.9)	85 (29.3)	7 (2.4)	35 (12.1%)
≥60	85	19 (22.4)	0 (0)	1 (1.2)	0 (0)	0 (0)	6 (7.1)	46 (54.1)	12 (14.1)	0 (0)	1 (1.2)
Negative	10285	605	747	121	64	16	575	4186	2071	381	1519
3-11	1004	220 (21.9)	720 (71.7)	64 (6.4)	0 (0)	0 (0)	0 (0)	0 (0)	0 (0)	0 (0)	0 (0)
12-17	792	13 (1.6)	25 (3.2)	14 (1.8)	5 (0.6)	6 (0.8)	151 (19.1)	578 (73)	0 (0)	0 (0)	0 (0)
18-59	7450	236 (3.2)	0 (0)	37 (0.5)	52 (0.7)	9 (0.1)	297 (4)	3017 (40.5)	1979 (26.6)	357 (4.8)	1466 (19.7)
≥60	1039	136 (13.1)	2 (0.2)	6 (0.6)	7 (0.7)	1 (0.1)	127 (12.2)	591 (56.9)	92 (8.9)	24 (2.3)	53 (5.1)

* Values in this row are time in days between the last vaccine dose and exposure to SARS-CoV-2.
